# Apigenin: a natural molecule at the intersection of sleep and aging

**DOI:** 10.3389/fnut.2024.1359176

**Published:** 2024-02-27

**Authors:** Daniel J. Kramer, Adiv A. Johnson

**Affiliations:** Tally Health, New York, NY, United States

**Keywords:** apigenin, sleep, aging, metabolism, NAD^+^, CD38

## Abstract

NAD^+^, a pivotal coenzyme central to metabolism, exhibits a characteristic decline with age. In mice, NAD^+^ levels can be elevated via treatment with apigenin, a natural flavonoid that inhibits the NAD^+^-consuming glycoprotein CD38. In animal models, apigenin positively impacts both sleep and longevity. For example, apigenin improves learning and memory in older mice, reduces tumor proliferation in a mouse xenograft model of triple-negative breast cancer, and induces sedative effects in mice and rats. Moreover, apigenin elongates survival in fly models of neurodegenerative disease and apigenin glycosides increase lifespan in worms. Apigenin’s therapeutic potential is underscored by human clinical studies using chamomile extract, which contains apigenin as an active ingredient. Collectively, chamomile extract has been reported to alleviate anxiety, improve mood, and relieve pain. Furthermore, dietary apigenin intake positively correlates with sleep quality in a large cohort of adults. Apigenin’s electron-rich flavonoid structure gives it strong bonding capacity to diverse molecular structures across receptors and enzymes. The effects of apigenin extend beyond CD38 inhibition, encompassing agonistic and antagonistic modulation of various targets, including GABA and inflammatory pathways. Cumulatively, a large body of evidence positions apigenin as a unique molecule capable of influencing both aging and sleep. Further studies are warranted to better understand apigenin’s nuanced mechanisms and clinical potential.

## 1 Introduction

One of the great early developments in the fields of biochemistry and metabolism was the discovery and characterization of nicotinamide adenine dinucleotide (NAD). The foundational work on this molecule occurred in multiple distinct stages (1). In 1906, Arthur Harden and William John Young proposed the existence of a “cozymase,” a chemical factor stable at high temperatures that increased the rate of the fermentation reaction in yeast (2). The involvement of a cofactor was an essential first insight, but they could not theorize on this factor’s molecular structure and chemical behavior. In 1930, Hans von Euler and Karl Myrbäck determined that Harden and Young’s cozymase contained an adenine (the aromatic ring structure also present in DNA, ATP, Coenzyme A, and other essential molecules) (3), a reducing sugar group, and a phosphate group (4).

While the identification of NAD’s structure allowed for theorizing on its mechanistic behavior, it was not until 1936 that Otto Warburg (the preeminent German scientist and Nobel laureate) and Walter Christian demonstrated that cozymase/NAD engaged in redox chemistry by transferring hydrides (5). NAD^+^ (the oxidized form of NAD) serves as an oxidizing agent, and NADH (NAD^+^ after receiving a hydride) functions as a reducing agent. It was subsequently demonstrated that NAD^+^/NADH plays a significant role in many diverse biochemical reactions that require electron exchange, a common motif across all living systems, including glycolysis, interconversions between pyruvate and lactate and pyruvate and acetyl-CoA, β-oxidation, the citric acid cycle, and oxidative phosphorylation (6). In addition, NAD^+^ can be chemically modified to create other molecules of use, including phosphorylation of NAD^+^ to NADP^+^ by NAD^+^ kinases (7), as well as conversion of NAD^+^ to ADP-ribose (ADPR) and cyclic ADP-ribose (cADPR) by the glycoprotein and ecto-enzyme CD38 (8). The presence and functions of NAD^+^ are conserved across all living cells, are associated with healthy and optimal function by biochemical and clinical measures, and both physical decline and aging correlate with lower NAD^+^ levels in humans (9).

The enzyme CD38 is a significant consumer of NAD^+^ and therefore, an essential regulator of its ambient concentrations and availability as a cofactor and substrate. CD38 was originally discovered in 1980 by Reinherz et al. (10). The finding was made as part of a larger study characterizing the surface of the T cell using monoclonal antibodies, which led to the identification of not only CD38 but also CD4, CD8, CD71/TFR-1, and others of varied functions, and CD38 was subsequently used as a marker of T cell identity (11, 12). Significant advancement in characterizing the biology of CD38 would come in 1992, when it was found to also be a glycoprotein cell surface marker on B cells, monocytes, bone marrow progenitors, and natural killer cells (13) and when experiments determined it to not only be a cell marker but a stimulator of activity in T and B cells (14).

**FIGURE 1:**
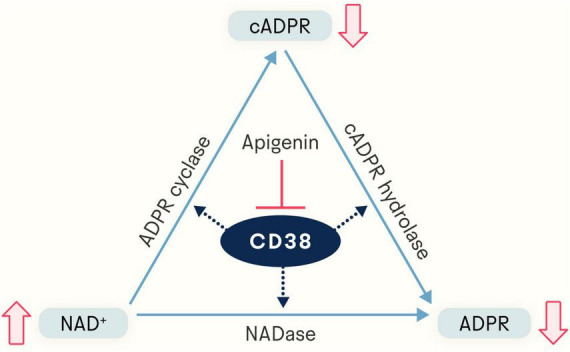
Apigenin indirectly elevates NAD^+^ levels via inhibition of CD38, a glycoprotein expressed in a variety of immune cells. The NADase CD38 generates both ADP-ribose (ADPR) and cyclic ADP-ribose (cADPR). By inhibiting CD38, apigenin increases the available pool of NAD^+^.

As to what activity and its role in it, there soon came evidence that CD38 has dual enzymatic functions, catalyzing both the conversion of NAD^+^ to cADPR as an ADP-ribosyl cyclase and the degradation of cADPR to ADPR as a cADPR-hydrolase (14, 15) (Figure 1). Under physiological conditions, 97% of the total output of CD38 is ADPR and the remaining 3% is the intermediate cADPR (16). Thus, CD38 requires a large amount of NAD^+^ to generate a pool of cADPR. It was also shown that the locations and membrane orientation of CD38 vary widely; it is present in the plasma membrane facing both the extracellular milieu and the intracellular cytosol, in a soluble form in the cytosol, as well as in the nuclear and mitochondrial membranes, allowing its consumption of NAD^+^ and production of cADPR and ADPR to influence extracellular and intracellular activities (17, 18).

While the quantity of cADPR produced is relatively small, it is still sufficient to contribute to cADPR’s known role in calcium signaling (19). cADPR increases calcium-induced calcium release at lower cytosolic concentrations of Ca^2+^ by targeting the Ca^2+^ uptake mechanism of the endoplasmic reticulum (20, 21). cADPR activates ryanodine receptors to initiate the release of calcium stored in the endoplasmic reticulum (21, 22). ADPR, the dominant product of CD38’s consumption of NAD^+^, is a strong synergistic agonist of the TRPM2 ion channel (23, 24). TRPM2, a calcium channel with connections to multiple age-related diseases (25, 26), is agonized by cADPR much more weakly than ADPR (24).

Given the many other more efficient means of regulating calcium signaling present in the cell, it has been theorized that the evolutionary role of CD38 is not to make cADPR and ADPR, but to deplete intracellular and extracellular NAD^+^. In this paradigm, cADPR and ADPR are just byproducts of NAD^+^ depletion (17). This would make CD38 not an ADP-ribosyl cyclase *per se* but instead an NAD^+^ glycohydrolase (27). Many studies have shown that high levels of CD38 and low levels of NAD^+^ are associated with aging, immunity, metabolic health, and cancer development (17, 28).

Given the biological significance of NAD^+^ and its regulator CD38, the discovery of the natural flavonoid 4’,5,7-trihydroxyflavone (C_15_H_10_O_5_), known as apigenin, and its characterization not only as an inhibitor of CD38 (thereby increasing levels of NAD^+^ and decreasing levels of cADPR and ADPR) (Figure 1) but also as a broadly-acting molecule (29) with effects on sleep and aging holds therapeutic potential in humans. In this review, we provide an in-depth description of apigenin, diving into mechanistic evidence as well as its ability to impact health in animal models and humans.

## 2 Biosynthesis, biochemistry, and bioavailability

Apigenin is a flavone-class aglycone, a glycoside without its glycosyl group, of several natural glycosides. Flavones are one of several sub-groups of flavonoids, the largest group of naturally occurring polyphenols that also include anthocyanidins, flavanols, flavanones, flavonols, and isoflavonoids (30, 31). Biosynthesis of apigenin only occurs in certain plants and, broadly speaking, flavonoids inhibit the transport of auxin, a morphogen-like plant hormone controlling growth and development (30).

**FIGURE 2:**
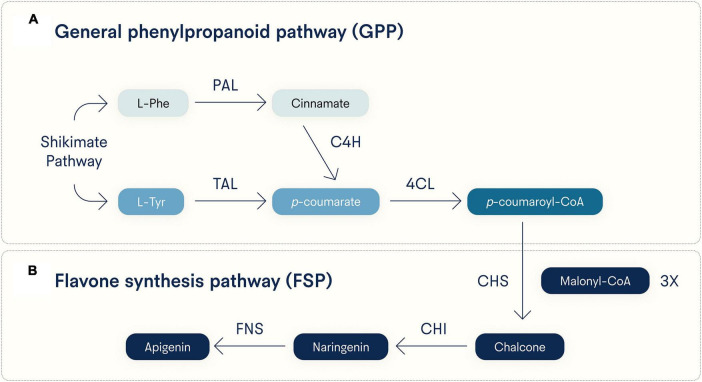
Major biosynthetic pathways for apigenin. Apigenin is synthesized in plants from either L-phenylalanine (L-Phe) or L-tyrosine (L-Tyr), both generated by the Shikimate pathway. Early and distinct reactive steps for each substrate converge on the last step of the **(A)** general phenylpropanoid pathway (GPP) and **(B)** a shared flavone synthesis pathway (FSP). Note that three equivalents of malonyl-CoA are incorporated into *p*-coumaroyl-CoA by CHS to generate chalcone. PAL, phenylalanine ammonia lyase; TAL, tyrosine ammonia lyase; C4H, cinnamate 4-hydroxylase; 4CL, 4-coumarate CoA ligase; CHS, chalcone synthase; CHI, chalcone isomerase; FNS, flavone synthase (soluble FNS1 and membrane-bound FNS2 forms).

As to its biosynthesis, apigenin can be generated via either of two converging pathways (Figure 2). One pathway starts with L-phenylalanine (L-Phe) and the other with L-tyrosine (L-Tyr), both products of the Shikimate pathway, a reactive process not found in animals but is used by plants to synthesize aromatic amino acids (32). Starting from L-Phe, it is converted to cinnamate via deamination by phenylalanine ammonia lyase (PAL), then oxidized by cinnamate 4-hydroxylase (C4H) to produce *p*-coumarate, which can also be made directly from L-tyrosine (L-Tyr) via deamination by tyrosine ammonia lyase (TAL) (33). The compound *p*-coumarate is then given a coenzyme A (CoA) at its carboxy group by 4-coumarate CoA ligase (4CL) to make *p-*coumaroyl-CoA. These two pathways toward *p*-coumaroyl-CoA synthesis comprise the general phenylpropanoid pathway (GPP, Figure 2A), and *p*-coumaroyl-CoA then enters the flavone synthesis pathway (FSP, Figure 2B) (33). In the FSP, chalcone synthase (CHS) uses three equivalents of malonyl-CoA to convert one *p*-coumaroyl-CoA to one chalcone. Chalcone isomerase (CHI) converts chalcone to naringenin, and finally, flavone synthase (either the soluble FNS1 or membrane-bound FNS2) oxidizes naringenin to apigenin (33).

The resulting product has a flavane nucleus of 15 carbon atoms (C6-C3-C6) and is considered a diphenyl-propanoid (34). The carbon atoms are arranged into two fused rings, the first of which is an oxygen-containing heterocycle and the second of which is a benzene ring constituting a phenylchromane nucleus, and an additional phenyl group is attached to the phenylchromane base (34). The molecule’s multiple rings, ketone group, and multiple hydroxyl groups create an electron-rich structure with a diverse assortment of binding affinities. Among other chemical feats, apigenin can scavenge hydroperoxide, chelate positive ions, and inhibit xanthine oxidase (35). Scavenging free radicals may be particularly important in apigenin’s putative antiviral, anti-inflammatory, and antimutagenic properties (30, 36).

Of the flavonoids synthesized by plants, apigenin is one of the most commonly found across species. Apigenin is present, mainly in a glycosylated form, in several vegetables, herbs, beverages, and fruit, including parsley, celery, onions, chamomile, thyme, oregano, basil, tea, beer, wine, grapefruit, and oranges (37). Aside from the apigenin glycosides (the most common of which is apigenin-7-O-glucoside), there are also glucuronide, acetylated, and methyl ester forms, acylated varieties, as well as monomers, dimers, and larger polymers (34, 38). The apigenin glycoside and its by-products are more water soluble than unmodified apigenin, and the glycosidic form may have a higher bioavailability (36). As a polyphenol, apigenin is part of a class of molecules that are mainly absorbed in the small intestine; here, 5–10% of the total ingested quantity of apigenin is absorbed, mostly in monomeric or dimeric forms (39). A total of 90–95% of the remaining apigenin that is unabsorbed makes its way to the colon up to millimolar levels (40, 41). Metabolism of apigenin occurs in the liver and “phase one” metabolism utilizes cytochrome P450 (enzymes that metabolize many different drugs) and nicotinamide adenine dinucleotide phosphate and flavin-containing monooxygenase (29, 40, 42) to generate monohydroxy derivatives (41). “Phase two” (conjugative) metabolism conjugates these derivatives and requires UDP-glucuronosyltransferases (41), enteric and enterohepatic cycling (43), glucuronidation and sulfation (29), and conversion into luteolin and sulfated and glucuronidated conjugates (44, 45).

In the intestinal lumen of the colon, apigenin is significantly metabolized by select species of microbiota adapted to utilize polyphenolic substrates (46, 47). This microbial activity converts polyphenols into smaller phenolic products (48, 49) that can be absorbed by the colon into circulation, and these metabolites may contribute to the health benefits ascribed to polyphenol intake, alongside the benefits of a given polyphenol (50, 51). Indeed, while 90–95% of ingested apigenin bypasses absorption in the small intestine and travels to the colon, animal studies have shown that less than 30% of the originally ingested quantity of apigenin is ultimately excreted in the feces, suggesting that a very significant portion of the total apigenin is metabolized by the microbiome into other molecules, which is indicative of physiological relevance (43, 52).

## 3 Apigenin and sleep

### 3.1 Animal models

Numerous studies across multiple animal models suggest that apigenin has connections with sleep and/or with its underlying neurobiology (Table 1). The invertebrate model *Drosophila melanogaster* can be used to study aspects of Parkinson’s disease (PD), a neurodegenerative disease characterized by brain aggregates of alpha-synuclein and disrupted sleep (53). In a 2017 study, an alpha-synuclein-expressing transgenic *D. melanogaster* model of PD was used to assess the effects of dietary supplementation with apigenin on PD symptoms and biomarkers. Apigenin- supplemented animals demonstrated a reversal of many biochemical changes characteristic of PD, including alterations in glutathione-*S*- transferase activity and lipid peroxidation as well as levels of glutathione and oxidative stress (54). Findings germane to oxidative stress are intriguing given that, in flies, sleep deprivation causes early death via the accumulation of reactive oxygen species (55). Apigenin was also reported to decrease activity of monoamine oxidase and increase levels of dopamine (54). It would be interesting to know if this finding is reproducible in normal flies and in more complex rodent models. In rodents, supplementation with apigenin increased sedation or reduced locomotor activity in rodents (56, 57), factors that influence sleep latency. Similar experiments in mice also suggested that apigenin can protect against neurodegeneration while improving learning, memory, neurovascular activity, and levels of several key biomarkers, including Bdnf, Trkb, and Creb, all of whose levels are influenced by sleep quality (58).

**TABLE 1:** Animal studies where an intervention involving apigenin positively influenced sleep and/or aging.

Organism	Intervention	Effect(s)	Mechanistic Finding(s)	References
Worm	Liquid medium containing 200 or 400 μg/mL apigenin glycosides until death	Extended lifespan and enhanced oxidative stress resistance	Reduced levels of reactive oxygen species Regulated the expression of several genes, including *daf-2, daf-16, sod-3, hsp-16.2, skn-1, gst-4, gcs-1, jnk-1*, and *sir-2.1* Promoted the translocation of daf-16 and skn-1 into the nucleus	(93)
	Diet containing 20, 40, or 80 μM apigenin until death	Induced a mitohormetic response and prolonged life	Reduced ATP production by transiently inhibiting mitochondrial respiration and temporarily increasing reactive oxygen species, triggering increased mitohormesis and an adaptive increase in antioxidant capacity Lifespan extension by apigenin was mediated by *aak-2*, *daf-16*, and *skn-1*	(94)
Fly	Diet containing 10 μM, 20 μM, 40 μM, or 80 μM apigenin for 24 days	Elongated lifespan and improved biochemical parameters in Parkinson’s disease flies	Increase in glutathione and dopamine levels Dose-dependent decreases in lipid peroxidation and activities of glutathione-*S*-transferase, monoamine oxidase, caspase-3, and caspase-9	(54)
	Treatment with 40 μM or 80 μM apigenin for seven days	Boosted lifespan and restored acetylcholinesterase activity in D-galactose-treated flies	Reduced oxidative stress Reduced opening of the mitochondrial permeability transition pore Lowered the activity of caspases 9 and 3	(92)
	Diet containing 25 μM, 50 μM, 75 μM, or 100 μM apigenin for 30 days	Preserved climbing ability and decreased oxidative stress in Alzheimer’s disease flies	Decreased oxidative stress and inhibited acetylcholinesterase Prevented the formation of amyloid beta aggregates	(70)
Mouse	Intraperitoneal injection of 50 mg/kg apigenin three hours before lethal injection with lipopolysaccharide	Increases survival in mice treated with high doses of lipopolysaccharide	Inhibited production of proinflammatory cytokines Il-1β, Il-8, and Tnf in lipopolysaccharide-stimulated mouse macrophages Non-canonical regulation of NF-κB activity *in vitro*	(95)
	Subcutaneous injection of 0.78 mg/kg apigenin for 10 days	Improved cardiometabolic health and thyroid parameters in mice with diabetes	Increased levels of insulin and thyroid hormone Reduced levels of glucose and cholesterol	(96)
	Oral administration of 1.5 mg/kg apigenin before 3 h of observation	Produced a mild sedative effect that was short-term	Locomotor activity was significantly lowered between 90 and 120 min after administration	(56)
	Oral administration of 10 mg/kg or 20 mg/kg apigenin daily for eight days	Improved learning and memory in mice with amyloid beta-induced amnesia	Protected neurovascular coupling, decreased neurovascular oxidative damage, and enhanced cerebral blood flow Blocked Acetylcholinesterase activity and increased acetylcholine levels Partially reversed the amyloid beta-driven decrease in Bdnf, TrkB, and phospho-CREB levels	(58)
	Intraperitoneal injection of 100 mg/kg apigenin for seven days	Elevated NAD^+^ levels and enhanced various aspects of sugar and lipid metabolism in obese animals	Inhibited the NADase Cd38 and augmented hepatic NAD^+^ levels Decreased global protein acetylation Improved glucose and lipid homeostasis	(97)
	Intraperitoneal injection of 20 or 40 mg/kg apigenin daily for 21 days	Lowered immobility time in depressive-like mice	Decreased the level of corticosterone while elevating the amount of Bdnf	(73)
	Intraperitoneal injection of 10 or 20 mg/kg apigenin daily for 20 days	Prevented behavioral and cognitive impairments in a mouse model of seizures	Increased levels of Bdnf, Creb, and phosphorylated Creb in the hippocampus Elevated level of serotonin Did not significantly affect seizure severity	(74)
	Oral administration of 0.5 mg/ml of apigenin daily for 42 days	Downregulated inflammatory genes and enhanced both learning and memory	Induced transcriptional changes linked to immunity, inflammation, and cytokine regulation Reduced inflammation and senescence in astrocytes	(101)
	Oral administration of 25 mg/kg of apigenin daily for 28 days	Restored heart function and inhibited cardiac inflammation in a mouse model of cardiomyopathy	In the heart, enhanced the transcription of genes related to the mitochondrial unfolded protein response	(99)
	Oral administration of five or 50 mg/kg apigenin for 56 days	Ameliorated atherosclerosis and improved metabolic parameters in a mouse model of atherosclerosis and non-alcoholic fatty liver disease	Reduced body weight and lowered plasma lipid levels Protected against the upregulation of inflammatory genes and pathways	(100)
	Intraperitoneal injection of 25 mg/kg apigenin daily for 28 days	Reduced tumor proliferation in a moues model of triple-negative breast cancer	Induced widespread transcriptional changes associated with alternative splicing Elevated apoptosis in tumor cells	(102)
Rat	Intraperitoneal injection of 25 mg/kg 15 min before pharmacological tests	Induced a sedative effect characterized by reduced locomotor activity	Observed effect was not mediated by an interaction with GABA-benzodiazepine receptors	(57)
	Oral administration of 75 mg/kg apigenin for 14 days	Protected diabetic animals against myocardial infarction	Reduced activity of creatine kinase and lactate dehydrogenase Decreased cell death and oxidative stress Increased expression of myocardial Pparg	(98)
	Intraperitoneal injection of 10 or 20 mg/kg apigenin for 14 days	Improved behavioral and biochemical signatures in a rat model of Parkinson’s disease	Decreased levels of Tnf-α, Il-6, and iNos1 Maintained mRNA levels of *Bdnf* and *Gdnf*	(77)

### 3.2 Clinical studies

Clinical studies assessing apigenin’s potential effects on sleep are promising, albeit limited (Table 2). Many of these studies utilize chamomile extract, which is often about 1% apigenin by mass (0.8–1.2%) and in which apigenin is a bioactive ingredient. It is difficult to be certain whether the effects observed from chamomile extract in these studies are solely due to apigenin, other components of chamomile, or a combination of both. However, these results, combined with the animal data described above, suggest an important role for apigenin.

**TABLE 2:** Human clinical trials involving apigenin-containing chamomile.

Subject Status	Intervention	Clinical Finding(s)	References
Primary insomnia	540 mg chamomile extract	Trended toward an improvement in daytime functioning	(59)
Generalized anxiety disorder with or without comorbid depression	220–1100 mg chamomile extract	Improvements in depression and mood scores	(60)
Postnatal with poor sleep quality	Chamomile tea	Scores for symptoms of depression and physical-symptoms-related sleep inefficiency were lowered	(64)
Generalized anxiety disorder	1500 mg chamomile extract	Attenuation of anxiety symptoms	(63)
Migraine without aura	2 ml of topical chamomile oleogel	Reductions in nausea, pain, phonophobia, vomiting, and photophobia	(66)
Generalized anxiety disorder with or without comorbid depression	1500 mg chamomile extract	Amelioration of anxiety	(62)

One study testing the effects of 270 mg of chamomile extract versus placebo twice per day for 28 days in patients with primary insomnia observed a trend toward improvement in daytime functioning, though it did not reach statistical significance (59). Another trial assessed the impact of chamomile extract versus placebo for eight weeks in patients with depression with or without anxiety. Subjects took a single 220 mg capsule daily in the first week and gradually increased this to five capsules (1100 mg) per day for the last four weeks. The authors observed significant reductions in depression and mood scores (60). While not measuring sleep directly, these mental health components are known contributors to sleep latency and quality, and vice versa (61). Additional studies have been conducted on multiple timescales assessing the potential of chamomile to treat anxiety. In one study, 1500 mg chamomile extract was given daily for eight weeks to two groups of patients, one with generalized anxiety disorder (GAD) and the other with GAD and comorbid depression. Chamomile extract reduced anxiety levels in both groups and attenuated depressive symptoms in the comorbid depression group (62). In a longer-term study, 500 mg chamomile extract was given three times daily for 12 weeks to patients with a primary diagnosis of moderate-to-severe GAD. Those whose GAD symptoms were ameliorated in response to treatment were then split into continued treatment versus placebo groups for 26 weeks. In addition to showing improved GAD symptoms during follow-up, chamomile-treated subjects also displayed significant reductions in body weight and mean arterial blood pressure (63).

Drinking chamomile tea after birth for two weeks also caused significant improvements in sleep efficiency and postnatal depression (64). These benefits largely disappeared four weeks post-test, though this could be due to broader changes in physiological activity weeks after birth, rather than a loss in efficacy of chamomile itself (65). Further, a topical formulation of chamomile containing chamazulene (an aromatic compound found in chamomile and related species) and apigenin was tested as a pain relief treatment in patients with migraine (66, 67), which can be a driver of poor sleep (67). Topical chamomile was found to significantly reduce pain, vomiting, nausea, phonophobia, and photophobia in patients with migraines (66). The analgesic properties may in part, be due to its reported ability to inhibit iNOS, PGE2, and COX2 (68). Further evidence comes from a recent cross-sectional study, which sought to identify associations between dietary levels of individual polyphenols and sleep quality. The flavonoid polyphenols apigenin and naringenin were both found to be significantly correlated with sleep quality. Specifically, a low level of dietary apigenin intake was associated with worse sleep quality (69). It would be interesting to better understand which of these effects are due to apigenin on its own or apigenin in conjunction with other molecules in chamomile.

### 3.3 Mechanistic evidence

Mechanistic studies suggest several mechanisms by which apigenin could increase sleep quality and quantity (Table 1). In *Drosophila*, dietary supplementation with apigenin decreased oxidative stress and acetylcholinesterase activity (70), reduced glutathione-*S*- transferase activity and lipid peroxidation, and increased glutathione levels, all of which are thought to support healthy sleep (71, 72). Apigenin supplementation in rodents decreased corticosterone levels and increased levels of Bdnf (73), CREB, phosphorylated CREB, and serotonin in the hippocampus (74, 75). These findings support the hypothesis of a pro-sleep role for apigenin, as the stress response is known to impair sleep (76). Moreover, BDNF, CREB, phosphorylated CREB (phospho-CREB), and serotonin have all been shown to be sleep-promoting, and BDNF, phospho-CREB, ACh, and TrkB were all found to be increased after apigenin treatment in a mouse model of amyloid beta (Aβ)-driven neurodegenerative disease (58).

In rats, apigenin demonstrated GABAergic activity that was independent of GABA- benzodiazepine receptors and possibly mediated by the GABA_*A*_ receptor (57). Apigenin also decreased levels of Tnf-α, Il-6, and iNos1 while maintaining elevated levels of Bdnf and glial Gdnf mRNA (77, 78). Findings involving BDNF are interesting given that this protein was reported to be significantly lower in insomniac patients with short sleep duration (79). Reports of apigenin ameliorating inflammatory markers are similarly intriguing, given a recent study showing that protracted sleep deprivation leads to severe inflammation in the mouse brain (80). While these studies suggest a prominent link between apigenin and sleep biomarkers, very little research has been done explaining how apigenin causally regulates sleep-relevant pathways. As such, future research is warranted to better understand the mechanistic relationship between apigenin and sleep health.

### 3.4 Sleep and aging

Given the evidence described above, it is important to note the close connection and interdependence between aging and sleep. Poor sleep can accelerate aging, as indicated by its alteration of age-associated epigenetic biomarkers (81) and its strong correlation with mortality risk (82) and healthspan (83). In turn, aging often leads to reduced sleep quality (84), producing a vicious cycle between the two. However, if poor sleep accelerates aging, which further worsens sleep, then improved sleep could improve aging, which would mitigate sleep’s decline. Thus, the interdependence between sleep and aging may complicate our understanding of apigenin’s effects. Longevity benefits reported in animal models could, for example, be due in part to improvements in sleep, a well-established reparative process. While this may be true, a closer look at the mechanistic data reported suggests that apigenin can directly mitigate established hallmarks of aging. Apigenin’s ability to act on a diversity of targets and processes makes it more likely that aging and sleep are largely being independently influenced.

At least some component of apigenin’s efficacy may be due to apigenin-derived metabolites generated by the intestinal microbiome, wherein different products may each contribute to specific elements of apigenin’s effects once entered into circulation. The microbiota has well-described roles in healthy sleep and aging, and dysbiosis is a known contributor to declines in both (85, 86). Indeed, dysbiosis is one of the 12 formally recognized hallmarks of aging (87). Changes in microbial composition could also at least partially explain the interdependence of sleep and aging, alongside other key factors like epigenetics and signaling molecules. Poor sleep quality and aging both alter distributions of microbial species, which alter the distributions of the end products of microbial chemistry and their resulting effects (88, 89). In addition, dietary supplementation with probiotics and targeted lifestyle modifications aimed at improving intestinal flora have been reported to improve sleep quality and mitigate immune and inflammatory aspects of the aging phenotype (90). While it’s clear that sleep can influence aging and vice versa, additional research is needed to understand this relationship on a deeper level. Moreover, it would be intriguing to learn if apigenin’s ability to influence aging (described in detail below) is due, at least in part, to effects on sleep.

## 4 Apigenin and aging

### 4.1 Animal models

Numerous research studies have examined the ability of apigenin to impact lifespan and/or age-related disease (Table 1). One model in *Drosophila* employs D-galactose, a compound that accelerates aging, shortens lifespan, and increases both oxidative stress and lipid peroxidation (91). In this model, apigenin partially reversed the lifespan-shortening effects of D-galactose (92). In a separate transgenic *Drosophila* model of Alzheimer’s disease (AD) expressing Amyloid Beta-42 in neurons and producing AD-like amyloid beta aggregates, a daily diet containing apigenin for 30 days was shown to delay the loss of climbing typically seen in this model (70). Apigenin similarly increases survival in a fly model of PD (54) and, in *Caenorhabditis elegans*, dietary supplementation with apigenin or apigenin glycosides increases lifespan (93, 94).

Promising results have also been documented in vertebrate animal models. In mice, intraperitoneal injection of apigenin before lipopolysaccharide injection strongly blunts the pro-inflammatory and immunomodulatory effects of lipopolysaccharide that are otherwise lethal at high doses (95). In diabetic mice, subcutaneous injection of apigenin improved cardiometabolic health and thyroid function (96). Intraperitoneal injection of apigenin improved glucose and lipid homeostasis in obese mice (97). Further, a diet supplemented with apigenin restored cardiac function and reduced the risk for isoproterenol-induced myocardial infarction in rats with streptozotocin-induced diabetes (98). In a separate mouse model of cardiomyopathy, apigenin delivered via gavage improved heart function and suppressed cardiac inflammation (99). Similarly, in a mouse model of atherosclerosis and non-alcoholic fatty liver disease, dietary apigenin reduced atherosclerosis, hepatic lipid accumulation, body weight, and plasma lipid levels (100). In addition, giving older mice apigenin in drinking water was shown to downregulate genes associated with immune activation and improve both learning and memory (101). In a recent study using a mouse xenograft model of triple-negative breast cancer, daily intraperitoneal injection of apigenin increased apoptosis in tumor cells and reduced tumor proliferation (102).

### 4.2 Clinical studies

Clinical studies have yet to directly investigate apigenin’s ability to influence aging. Some studies have assessed apigenin more directly, though mostly in connection to sleep, depression, pain, and anxiety, as described previously (Table 2). While sleep and mental health quality are significant contributors to aging and health (84, 103), human studies assessing apigenin’s potential effect on established biomarkers of aging, such as markers of cardiometabolic health, motor function, and cognition, are certainly needed. Some clinical studies have utilized other flavonoids chemically similar to apigenin, such as quercetin. With the exception of quercetin’s two additional hydroxyl groups, quercetin and apigenin are chemically identical (104). Other research has employed wider varieties of non-flavonoid polyphenols, including phenolic acids, lignans, and stilbenes (105), and many molecules across the flavonoid and non-flavonoid polyphenol classes have been reported to target hallmarks of aging, including cellular senescence (104).

As described heretofore, one of apigenin’s better-characterized functions is to increase levels of NAD^+^ via inhibition of the NADase enzyme CD38. Specifically, mouse NAD^+^ levels in the liver were nearly doubled in response to apigenin (97). The role of NAD^+^ in health and longevity is beyond the scope of this review but has been comprehensively described elsewhere (106). Previous work has shown that NAD^+^ levels decline with age (107), including in specific tissues such as the brain (108). This is due in part to increased consumption and decreased production and salvage of NAD^+^ (109, 110). Older humans with higher levels of NAD^+^ relative to their age group tend to be healthier and exhibit fewer age-associated disease phenotypes (111). While NAD^+^ levels often decline with age in human skeletal muscle, exercise-trained older individuals display NAD^+^ levels comparable to younger subjects (112).

Clinical studies indicate that NAD^+^ levels can be modulated by behavior change and/or supplementation. In humans and mice, exercise of moderate intensity and resistance training appears to increase levels of circulating NAD^+^, NADH, and NAMPT (113, 114), providing one of several possible biochemical explanations linking exercise to healthy aging. Direct supplementation in humans with NAD^+^ precursors may also offer benefits. Analogs of nicotinic acid, an NAD^+^ precursor also known as niacin or vitamin B3, improved mitochondrial function in skeletal muscle in patients with diabetes (115). Nicotinamide mononucleotide, a precursor to NAD^+^, was reported to be safe and well-tolerated (116), increase NAD^+^ levels (117), augment insulin sensitivity in muscle (118), enhance energy (119), and reduce blood pressure (120). Nicotinamide mononucleotide’s effects were found to be more mixed on grip strength (117, 121) and walking ability (121, 122). Nicotinamide riboside, another NAD^+^ precursor reported to be safe and well-tolerated (123), was similarly shown to increase levels of NAD^+^ (124). Nicotinamide riboside was also reported to decrease inflammation and enacted transcriptional changes pertinent to metabolism and mitochondrial function in healthy and overweight individuals (125). While exciting, larger, longer-term clinical trials are needed to better understand the safety and efficacy of direct NAD^+^ precursors like nicotinamide mononucleotide and nicotinamide riboside.

### 4.3 Mechanistic evidence

While apigenin may be positively influencing aging by elevating NAD^+^ levels, it could also have other systemic pro-longevity effects (Table 1). Apigenin mitigated D-galactose-driven accelerated aging in *Drosophila* by attenuating oxidative stress, reducing the opening of the mitochondrial permeability transition pore, and reducing the activity of caspases 9 and 3 (92). Concomitant with slowing disease progression in a transgenic fly model of AD, apigenin decreases oxidative stress, lowers acetylcholinesterase activity, and inhibits the formation of amyloid beta-42 aggregates (70). Long-lived worms treated with apigenin glycosides exhibited reduced reactive oxygen species levels and altered expression of numerous genes, including *daf-2, daf-16, sod-3, hsp-16.2, skn-1, gst-4, gcs-1, jnk-1*, and *sir-2.1.* Apigenin glycosides also promoted the translocation of daf-16 and skn-1 into the nucleus (93). In a more recent worm study, apigenin was shown to prolong lifespan by transiently inhibiting mitochondrial respiration and temporarily increasing reactive oxygen species levels. This, in turn, triggered a mitohormetic response which led to an overall increased capacity to deal with oxidative stress. Life extension also depended on the genes *aak-2*, *daf-16*, and *skn-1* (94). Such findings are interesting given that, in a mouse model of myocardial injury, the protective effects of apigenin were dependent on the mitochondrial unfolded protein response (99).

In lipopolysaccharide-stimulated mouse macrophages, apigenin inhibits the production of the proinflammatory cytokines Il-1β, Il-8, and Tnf. This flavonoid also engages in non-canonical regulation of NF-κB transcription factor activity through hypophosphorylation of Ser536 in the p65 subunit and the inactivation of the lipopolysaccharide-stimulated IKK complex (95). Apigenin has also been reported to improve glucose and lipid homeostasis in mice by increasing levels of insulin and thyroid hormone and reducing levels of glucose, glucose-metabolizing liver enzymes, and cholesterol (96). Further, *Cd38* knockout mice fed a high-fat diet have higher levels of NAD^+^, are less likely to develop obesity and metabolic syndrome, and have increased survival compared to wild-type animals (97). In contrast, *Parp1* knockout mice show worse survival on a high-fat diet. This may be due to the role Parp1 plays in DNA repair and genomic stability (97). Additionally, in *Ldlr* and *Nlrp3* knockout mice fed a high-fat diet, apigenin appeared to reverse the cardiac and hepatic symptoms of the *Ldlr*^–/–^ genotype in an inflammasome-dependent manner, as the apparent benefits of apigenin were abrogated in the double knockout, and treatment of liver cells cultured *in vitro* demonstrated consistent findings (100).

Apigenin’s cognitive effects may be mediated by specific cell types. Older mice given apigenin in drinking water experienced glial cell-associated transcriptional changes across immunity, inflammation, and cytokine regulation into expression profiles that were characteristic of younger animals, and they also exhibited reduced inflammation and cellular senescence in astrocytes (101). In a recent study using immuno-deficient mice implanted with human tumor cell xenografts, apigenin appeared to induce transcriptome-wide reprogramming of cancer-associated alternative splicing in an RNA binding protein (hnRNPA2)-associated manner and induce switching of cancer-associated to non-cancer-associated alternative spliced isoforms (102). Studies in rats also allude to potential mechanisms of action for apigenin. Work by Ogura et al showed that, in a rat model of diabetic kidney disease, apigenin ameliorated renal injuries, pro-inflammatory gene expression, and tubular cell damage. In the kidney, apigenin also elevated the NAD^+^/NADH ratio and enhanced the activity of Sirt3 (126). In rats, protein expression of the nuclear receptor Pparg in the myocardium was elevated by apigenin (98).

## 5 Conclusion and future directions

**FIGURE 3:**
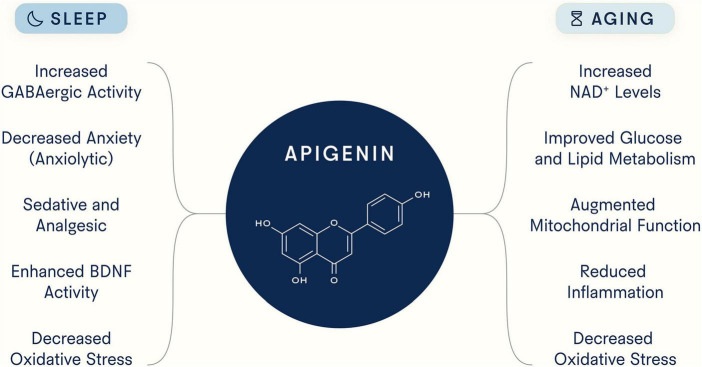
Potential mechanisms underlying apigenin’s ability to target sleep and aging. As a flavonoid with strong binding capacity to distinct molecular structures, apigenin has been reported to target myriad processes and pathways.

Although compelling evidence suggests that apigenin exhibits both pro-longevity and pro-sleep properties (Figure 3), many important questions remain. First, given the binding promiscuity of flavonoid polyphenols and apigenin’s broad systemic effects, additional work is needed to determine (1) whether there are additional targets of apigenin, (2) optimal dosage and safety over longer treatment periods and in different patient populations, and (3) the biological effects of all of apigenin’s known chemical derivatives and the extent to which each contributes to the health benefits described here. There are many chemical forms of apigenin, each with unique features and products, that could affect biology in different ways, and to date there has not been extensive work aimed at comparing them. For example, Elkhedir et al. specifically used apigenin 6-C-arabinoside-8-C-glucoside and apigenin 6,8-di-C-glucoside, the predominant derivates in green pepper (93). While this study found animals treated with these compounds experienced multiple longevity benefits, further work is needed to determine which of these benefits are unique to apigenin derivatives versus apigenin itself. In addition, the therapeutic effects of apigenin could potentially be enhanced by improving its bioavailability, given its low absorption rate in the small intestine. However, the potential benefits of increased absorption of apigenin in the small intestine must be weighed against the reduced availability of apigenin in the large intestine for microbial conversion to smaller phenolic metabolites, which, as stated earlier, are also absorbed into the circulation and could exert their own effects on sleep and aging.

Further, not all mechanisms of increasing NAD^+^ levels are similarly beneficial or effective. For example, elevating NAD^+^ levels by inhibiting CD38 - an immune cell glycoprotein - may be more desirable than elevating NAD^+^ levels by inhibiting PARP1 – an enzyme that responds to DNA damage and promotes DNA repair. Systematic comparisons and risk/benefit analyses of different interventions that boost NAD^+^ levels would be valuable. More clinical studies are also needed to assess the ability of apigenin on its own – as opposed to apigenin in chamomile extract – to impact sleep-relevant parameters. Lastly, additional research is warranted to illuminate apigenin’s mechanisms of action.

## Author contributions

DK: Conceptualization, Visualization, Writing−original draft, Writing−review and editing. AJ: Conceptualization, Visualization, Writing−original draft, Writing−review and editing.

## References

[1] Palmer R Elnashar M Vaccarezza M. Precursor comparisons for the upregulation of nicotinamide adenine dinucleotide. Novel approaches for better aging. *Aging Med.* (2021) 4:214–20. 10.1002/agm2.12170 34553119 PMC8444956

[2] Harden A Young W Martin C. The alcoholic ferment of yeast-juice. Part II.—The coferment of yeast-juice. *Proc R Soc Lond Ser B Cont Pap Biol Charact.* (1906) 78:369–75. 10.1098/rspb.1906.0070

[3] Denessiouk K Rantanen V Johnson M. Adenine recognition: a motif present in ATP-, CoA-, NAD-, NADP-, and FAD-dependent proteins. *Proteins.* (2001) 44:282–91. 10.1002/prot.1093 11455601

[4] Fessel J Oldham W. Pyridine Dinucleotides from Molecules to Man. *Antioxid Redox Signal.* (2018) 28:180–212. 10.1089/ars.2017.7120 28635300 PMC5737636

[5] Yang Y Sauve A. NAD(+) metabolism: Bioenergetics, signaling and manipulation for therapy. *Biochim Biophys Acta.* (2016) 1864:1787–800. 10.1016/j.bbapap.2016.06.014 27374990 PMC5521000

[6] Katsyuba E Auwerx J. Modulating NAD(+) metabolism, from bench to bedside. *EMBO J.* (2017) 36:2670–83. 10.15252/embj.201797135 28784597 PMC5599801

[7] Oka S Titus A Zablocki D Sadoshima J. Molecular properties and regulation of NAD(+) kinase (n.d.). *Redox Biol.* (2023) 59:102561. 10.1016/j.redox.2022.102561 36512915 PMC9763689

[8] Graeff R Liu Q Kriksunov I Kotaka M Oppenheimer N Hao Q Mechanism of cyclizing NAD to cyclic ADP-ribose by ADP-ribosyl cyclase and CD38. *J Biol Chem.* (2009) 284:27629–36. 10.1074/jbc.M109.030965 19640843 PMC2785691

[9] Covarrubias A Perrone R Grozio A Verdin E. NAD(+) metabolism and its roles in cellular processes during ageing. *Nat Rev Mol Cell Biol.* (2021) 22:119–41. 10.1038/s41580-020-00313-x 33353981 PMC7963035

[10] Reinherz E Kung P Goldstein G Levey R Schlossman S. Discrete stages of human intrathymic differentiation: analysis of normal thymocytes and leukemic lymphoblasts of T-cell lineage. *Proc Natl Acad Sci U S A.* (1980) 77:1588–92. 10.1073/pnas.77.3.1588 6966400 PMC348542

[11] Malavasi F Deaglio S Funaro A Ferrero E Horenstein A Ortolan E Evolution and function of the ADP ribosyl cyclase/CD38 gene family in physiology and pathology. *Physiol Rev.* (2008) 88:841–86. 10.1152/physrev.00035.2007 18626062

[12] Golden-Mason L Curry M Nolan N Traynor O McEntee G Kelly J Differential expression of lymphoid and myeloid markers on differentiating hematopoietic stem cells in normal and tumor-bearing adult human liver. *Hepatology.* (2000) 31:1251–6. 10.1053/jhep.2000.7713 10827150

[13] Hartman W Pelleymounter L Moon I Kalari K Liu M Wu T CD38 expression, function, and gene resequencing in a human lymphoblastoid cell line-based model system. *Leuk Lymphoma.* (2010) 51:1315–25. 10.3109/10428194.2010.483299 20470215 PMC2892000

[14] Ferrero E Malavasi FA. Natural History of the Human CD38 Gene. In: Lee H editor. *Cyclic ADP-Ribose and NAADP: Structures, Metabolism and Functions.* Boston, MA: Springer (2002). 10.1007/978-1-4615-0269-2_4

[15] Franco L Guida L Bruzzone S Zocchi E Usai C De Flora A. The transmembrane glycoprotein CD38 is a catalytically active transporter responsible for generation and influx of the second messenger cyclic ADP-ribose across membranes. *FASEB J.* (1998) 12:1507–20. 10.1096/fasebj.12.14.1507 9806760

[16] Chini E. CD38 as a regulator of cellular NAD: a novel potential pharmacological target for metabolic conditions. *Curr Pharm Des.* (2009) 15:57–63. 10.2174/138161209787185788 19149603 PMC2883294

[17] Hogan K Chini C Chini E. The Multi-faceted Ecto-enzyme CD38: Roles in Immunomodulation, Cancer, Aging, and Metabolic Diseases. *Front Immunol.* (2019) 10:1187. 10.3389/fimmu.2019.01187 31214171 PMC6555258

[18] Piedra-Quintero Z Wilson Z Nava P Guerau-de-Arellano M. CD38: An Immunomodulatory Molecule in Inflammation and Autoimmunity. *Front Immunol.* (2020) 11:597959. 10.3389/fimmu.2020.597959 33329591 PMC7734206

[19] Guse A. Biochemistry, biology, and pharmacology of cyclic adenosine diphosphoribose (cADPR). *Curr Med Chem.* (2004) 11:847–55. 10.2174/0929867043455602 15078169

[20] Lee H. Cyclic ADP-ribose and NAADP: fraternal twin messengers for calcium signaling. *Sci China Life Sci.* (2011) 54:699–711. 10.1007/s11427-011-4197-3 21786193

[21] Galione A Chuang K. Pyridine Nucleotide Metabolites and Calcium Release from Intracellular Stores. *Adv Exp Med Biol.* (2020) 1131:371–94. 10.1007/978-3-030-12457-1_15 31646518

[22] Santulli G Marks A. Essential roles of intracellular calcium release channels in muscle. Brain, metabolism, and aging. *Curr Mol Pharmacol.* (2015) 8:206–22. 10.2174/1874467208666150507105105 25966694

[23] Fonfria E Marshall I Benham C Boyfield I Brown J Hill K TRPM2 channel opening in response to oxidative stress is dependent on activation of poly(ADP-ribose) polymerase. *Br J Pharmacol.* (2004) 143:186–92. 10.1038/sj.bjp.0705914 15302683 PMC1575275

[24] Yu P Cai X Liang Y Wang M Yang W. Roles of NAD(+) and its metabolites regulated calcium channels in cancer. *Molecules.* (2020) 25:4826. 10.3390/molecules25204826 33092205 PMC7587972

[25] Belrose J Jackson M. TRPM2: a candidate therapeutic target for treating neurological diseases. *Acta Pharmacol Sin.* (2018) 39:722–32. 10.1038/aps.2018.31 29671419 PMC5943913

[26] Ali E Chakrabarty B Ramproshad S Mondal B Kundu N Sarkar C TRPM2-mediated Ca(2+) signaling as a potential therapeutic target in cancer treatment: an updated review of its role in survival and proliferation of cancer cells. *Cell Commun Signal.* (2023) 21:145. 10.1186/s12964-023-01149-6 37337283 PMC10278317

[27] Berthelier V Tixier J Muller-Steffner H Schuber F Deterre P. Human CD38 is an authentic NAD(P)+ glycohydrolase. *Biochem J.* (1998) 330:1383–90. 10.1042/bj3301383 9494110 PMC1219286

[28] van de Donk N Usmani S. CD38 antibodies in multiple myeloma: mechanisms of action and modes of resistance. *Front Immunol.* (2018) 9:2134. 10.3389/fimmu.2018.02134 30294326 PMC6158369

[29] Tang D Chen K Huang L Li J. Pharmacokinetic properties and drug interactions of apigenin, a natural flavone. *Expert Opin Drug Metab Toxicol.* (2017) 13:323–30. 10.1080/17425255.2017.1251903 27766890

[30] Falcone Ferreyra M Rius S Casati P. Flavonoids: biosynthesis, biological functions, and biotechnological applications. *Front Plant Sci.* (2012) 3:222. 10.3389/fpls.2012.00222 23060891 PMC3460232

[31] Kabera J Semana E Mussa A He X. Plant secondary metabolites: biosynthesis, classification, function and pharmacological properties. *J Pharm Pharmacol.* (2014) 2:377–92.

[32] Herrmann K. The shikimate pathway as an entry to aromatic secondary metabolism. *Plant Physiol.* (1995) 107:7–12. 10.1104/pp.107.1.7 7870841 PMC161158

[33] Forkmann G. Flavonoids as flower pigments: the formation of the natural spectrum and its extension by genetic engineering. *Plant Breeding.* (1991) 106:1–26. 10.1111/j.1439-0523.1991.tb00474.x

[34] Salehi B Venditti A Sharifi-Rad M Kregiel D Sharifi-Rad J Durazzo A The Therapeutic Potential of Apigenin. *Int J Mol Sci.* (2019) 20:1305. 10.3390/ijms20061305 30875872 PMC6472148

[35] Spiegel M Sroka Z. Quantum-mechanical characteristics of apigenin: Antiradical, metal chelation and inhibitory properties in physiologically relevant media. *Fitoterapia.* (2023) 164:105352. 10.1016/j.fitote.2022.105352 36400153

[36] Patel D Shukla S Gupta S. Apigenin and cancer chemoprevention: progress, potential and promise (review). *Int J Oncol.* (2007) 30:233–45. 10.3892/ijo.30.1.233 17143534

[37] Hostetler G Ralston R Schwartz S. Flavones: Food Sources, Bioavailability, Metabolism, and Bioactivity. *Adv Nutr.* (2017) 8:423–35. 10.3945/an.116.012948 28507008 PMC5421117

[38] Shankar E Goel A Gupta K Gupta S. Plant flavone apigenin: An emerging anticancer agent. *Curr Pharmacol Rep.* (2017) 3:423–46. 10.1007/s40495-017-0113-2 29399439 PMC5791748

[39] Manach C Williamson G Morand C Scalbert A Remesy C. Bioavailability and bioefficacy of polyphenols in humans. I. Review of 97 bioavailability studies. *Am J Clin Nutr.* (2005) 81(1 Suppl):230S–42S. 10.1093/ajcn/81.1.230S 15640486

[40] Cardona F Andres-Lacueva C Tulipani S Tinahones F Queipo-Ortuno M. Benefits of polyphenols on gut microbiota and implications in human health. *J Nutr Biochem.* (2013) 24:1415–22. 10.1016/j.jnutbio.2013.05.001 23849454

[41] Mushtaq Z Sadeer N Hussain M Mahwish, Alsagaby S Imran M. Therapeutical properties of apigenin: a review on the experimental evidence and basic mechanisms. *Int J Food Prop.* (2023) 26:1914–39. 10.1080/10942912.2023.2236329

[42] Ashrafizadeh M Bakhoda M Bahmanpour Z Ilkhani K Zarrabi A Makvandi P Apigenin as tumor suppressor in cancers: biotherapeutic activity, nanodelivery, and mechanisms with emphasis on pancreatic cancer. *Front Chem.* (2020) 8:829. 10.3389/fchem.2020.00829 33195038 PMC7593821

[43] Chen T Li L Lu X Jiang H Zeng S. Absorption and excretion of luteolin and apigenin in rats after oral administration of Chrysanthemum morifolium extract. *J Agric Food Chem.* (2007) 55:273–7. 10.1021/jf062088r 17227053

[44] Chen J Lin H Hu M. Metabolism of flavonoids via enteric recycling: role of intestinal disposition. *J Pharmacol Exp Ther.* (2003) 304:1228–35. 10.1124/jpet.102.046409 12604700

[45] Gradolatto A Canivenc-Lavier M Basly J Siess M Teyssier C. Metabolism of apigenin by rat liver phase I and phase ii enzymes and by isolated perfused rat liver. *Drug Metab Dispos.* (2004) 32:58–65. 10.1124/dmd.32.1.58 14709621

[46] Manach C Scalbert A Morand C Remesy C Jimenez L. Polyphenols: food sources and bioavailability. *Am J Clin Nutr.* (2004) 79:727–47. 10.1093/ajcn/79.5.727 15113710

[47] D’Archivio M Filesi C Di Benedetto R Gargiulo R Giovannini C Masella R. Polyphenols, dietary sources and bioavailability. *Ann Ist Super Sanita.* (2007) 43:348–61. 18209268

[48] Manach C Donovan J. Pharmacokinetics and metabolism of dietary flavonoids in humans. *Free Radic Res.* (2004) 38:771–85. 10.1080/10715760410001727858 15493450

[49] Serrano J Puupponen-Pimia R Dauer A Aura A Saura-Calixto F. Tannins: current knowledge of food sources, intake, bioavailability and biological effects. *Mol Nutr Food Res.* (2009) 53:S310–29. 10.1002/mnfr.200900039 19437486

[50] Appeldoorn M Vincken J Aura A Hollman P Gruppen H. Procyanidin dimers are metabolized by human microbiota with 2-(3,4-dihydroxyphenyl)acetic acid and 5-(3,4-dihydroxyphenyl)-gamma-valerolactone as the major metabolites. *J Agric Food Chem.* (2009) 57:1084–92. 10.1021/jf803059z 19191673

[51] Deprez S Brezillon C Rabot S Philippe C Mila I Lapierre C Polymeric proanthocyanidins are catabolized by human colonic microflora into low-molecular-weight phenolic acids. *J Nutr.* (2000) 130:2733–8. 10.1093/jn/130.11.2733 11053514

[52] Gradolatto A Basly J Berges R Teyssier C Chagnon M Siess M Pharmacokinetics and metabolism of apigenin in female and male rats after a single oral administration. *Drug Metab Dispos.* (2005) 33:49–54. 10.1124/dmd.104.000893 15466493

[53] Zuzuarregui J During E. Sleep Issues in Parkinson’s Disease and Their Management. *Neurotherapeutics.* (2020) 17:1480–94. 10.1007/s13311-020-00938-y 33029723 PMC7851262

[54] Siddique Y Jyoti S. Alteration in biochemical parameters in the brain of transgenic Drosophila melanogaster model of Parkinson’s disease exposed to apigenin. *Integr Med Res.* (2017) 6:245–53. 10.1016/j.imr.2017.04.003 28951838 PMC5605376

[55] Vaccaro A Kaplan Dor Y Nambara K Pollina E Lin C Greenberg M Sleep loss can cause death through accumulation of reactive oxygen species in the gut. *Cell.* (2020) 181:1307–28e15. 10.1016/j.cell.2020.04.049 32502393

[56] Chow N Fretz M Hamburger M Butterweck V. Telemetry as a tool to measure sedative effects of a valerian root extract and its single constituents in mice. *Planta Med.* (2011) 77:795–803. 10.1055/s-0030-1250589 21154200

[57] Zanoli P Avallone R Baraldi M. Behavioral characterisation of the flavonoids apigenin and chrysin. *Fitoterapia.* (2000) 71:S117–23. 10.1016/S0367-326X(00)00186-6 10930722

[58] Liu R Zhang T Yang H Lan X Ying J Du G. The flavonoid apigenin protects brain neurovascular coupling against amyloid-beta(-)-induced toxicity in mice. *J Alzheimers Dis.* (2011) 24:85–100. 10.3233/JAD-2010-101593 21297270

[59] Zick S Wright B Sen A Arnedt J. Preliminary examination of the efficacy and safety of a standardized chamomile extract for chronic primary insomnia: a randomized placebo-controlled pilot study. *BMC Complement Altern Med.* (2011) 11:78. 10.1186/1472-6882-11-78 21939549 PMC3198755

[60] Amsterdam J Shults J Soeller I Mao J Rockwell K Newberg A. Chamomile (*Matricaria recutita*) may provide antidepressant activity in anxious, depressed humans: an exploratory study. *Altern Ther Health Med.* (2012) 18:44–9. PMC3600408 22894890

[61] Triantafillou S Saeb S Lattie E Mohr D Kording K. Relationship between sleep quality and mood: ecological momentary assessment study. *JMIR Ment Health.* (2019) 6:e12613. 10.2196/12613 30916663 PMC6456824

[62] Amsterdam J Li Q Xie S Mao J. Putative antidepressant effect of chamomile (*Matricaria chamomilla* L.) oral extract in subjects with comorbid generalized anxiety disorder and depression. *J Altern Complement Med.* (2020) 26:813–9. 10.1089/acm.2019.0252 31808709 PMC7488203

[63] Mao J Xie S Keefe J Soeller I Li Q Amsterdam J. Long-term chamomile (*Matricaria chamomilla* L.) treatment for generalized anxiety disorder: A randomized clinical trial. *Phytomedicine.* (2016) 23:1735–42. 10.1016/j.phymed.2016.10.012 27912875 PMC5646235

[64] Chang S Chen C. Effects of an intervention with drinking chamomile tea on sleep quality and depression in sleep disturbed postnatal women: a randomized controlled trial. *J Adv Nurs.* (2016) 72:306–15. 10.1111/jan.12836 26483209

[65] Achtyes E Keaton S Smart L Burmeister A Heilman P Krzyzanowski S Inflammation and kynurenine pathway dysregulation in post-partum women with severe and suicidal depression. *Brain Behav Immun.* (2020) 83:239–47. 10.1016/j.bbi.2019.10.017 31698012 PMC6906225

[66] Zargaran A Borhani-Haghighi A Salehi-Marzijarani M Faridi P Daneshamouz S Azadi A Evaluation of the effect of topical chamomile (*Matricaria chamomilla* L.) oleogel as pain relief in migraine without aura: a randomized, double-blind, placebo-controlled, crossover study. *Neurol Sci.* (2018) 39:1345–53. 10.1007/s10072-018-3415-1 29808331

[67] Lin Y Lin G Lee J Lee M Tsai C Hsu Y Associations between sleep quality and migraine frequency: a cross-sectional case-control study. *Medicine.* (2016) 95:e3554. 10.1097/MD.0000000000003554 27124064 PMC4998727

[68] Zargaran A Borhani-Haghighi A Faridi P Daneshamouz S Kordafshari G Mohagheghzadeh A. Potential effect and mechanism of action of topical chamomile (*Matricaria chammomila* L.) oil on migraine headache: a medical hypothesis. *Med Hypotheses.* (2014) 83:566–9. 10.1016/j.mehy.2014.08.023 25238714

[69] Godos J Ferri R Castellano S Angelino D Mena P Del Rio D Specific dietary (poly)phenols are associated with sleep quality in a cohort of italian adults. *Nutrients.* (2020) 12:1226. 10.3390/nu12051226 32357534 PMC7282005

[70] Siddique Y Rahul, Ara G Afzal M Varshney H Gaur K. Beneficial effects of apigenin on the transgenic Drosophila model of Alzheimer’s disease. *Chem Biol Interact.* (2022) 366:110120. 10.1016/j.cbi.2022.110120 36027948

[71] Watson C Baghdoyan H Lydic R. Neuropharmacology of sleep and wakefulness. *Sleep Med Clin.* (2010) 5:513–28. 10.1016/j.jsmc.2010.08.003 21278831 PMC3026477

[72] Wilking M Ndiaye M Mukhtar H Ahmad N. Circadian rhythm connections to oxidative stress: implications for human health. *Antioxid Redox Signal.* (2013) 19:192–208. 10.1089/ars.2012.4889 23198849 PMC3689169

[73] Weng L Guo X Li Y Yang X Han Y. Apigenin reverses depression-like behavior induced by chronic corticosterone treatment in mice. *Eur J Pharmacol.* (2016) 774:50–4. 10.1016/j.ejphar.2016.01.015 26826594

[74] Sharma P Sharma S Singh D. Apigenin reverses behavioural impairments and cognitive decline in kindled mice via CREB-BDNF upregulation in the hippocampus. *Nutr Neurosci.* (2020) 23:118–27. 10.1080/1028415X.2018.1478653 29847220

[75] Kalivarathan J Kalaivanan K Chandrasekaran S Nanda D Ramachandran V Carani Venkatraman A. Apigenin modulates hippocampal CREB-BDNF signaling in high fat, high fructose diet-fed rats. *J Funct Foods.* (2020) 68:103898. 10.1016/j.jff.2020.103898

[76] Nollet M Wisden W Franks N. Sleep deprivation and stress: a reciprocal relationship. *Interface Focus.* (2020) 10:20190092. 10.1098/rsfs.2019.0092 32382403 PMC7202382

[77] Anusha C Sumathi T Joseph L. Protective role of apigenin on rotenone induced rat model of Parkinson’s disease: Suppression of neuroinflammation and oxidative stress mediated apoptosis. *Chem Biol Interact.* (2017) 269:67–79. 10.1016/j.cbi.2017.03.016 28389404

[78] Wang L Gao Z Chen G Geng D Gao D. Low levels of adenosine and GDNF are potential risk factors for Parkinson’s disease with sleep disorders. *Brain Sci.* (2023) 13:200. 10.3390/brainsci13020200 36831743 PMC9953846

[79] Furihata R Saitoh K Otsuki R Murata S Suzuki M Jike M Association between reduced serum BDNF levels and insomnia with short sleep duration among female hospital nurses. *Sleep Med.* (2020) 68:167–72. 10.1016/j.sleep.2019.12.011 32044553

[80] Sang D Lin K Yang Y Ran G Li B Chen C Prolonged sleep deprivation induces a cytokine-storm-like syndrome in mammals. *Cell.* (2023) 186:5500–16e21. 10.1016/j.cell.2023.10.025 38016470

[81] Kusters C Klopack E Crimmins E Seeman T Cole S Carroll J. Short sleep and insomnia are associated with accelerated epigenetic age. *Psychosom Med.* (2023) [Epub ahead of print]. 10.1097/PSY.0000000000001243 37594243 PMC10879461

[82] Svensson T Saito E Svensson A Melander O Orho-Melander M Mimura M Association of sleep duration with all- and major-cause mortality among adults in Japan, China, Singapore, and Korea. *JAMA Netw Open.* (2021) 4:e2122837. 10.1001/jamanetworkopen.2021.22837 34477853 PMC8417759

[83] Sambou M Zhao X Hong T Fan J Basnet T Zhu M Associations between sleep quality and health span: a prospective cohort study based on 328,850 UK Biobank participants. *Front Genet.* (2021) 12:663449. 10.3389/fgene.2021.663449 34211497 PMC8239359

[84] Mander B Winer J Walker M. Sleep and human aging. *Neuron.* (2017) 94:19–36. 10.1016/j.neuron.2017.02.004 28384471 PMC5810920

[85] Holzhausen E Peppard P Sethi A Safdar N Malecki K Schultz A Associations of gut microbiome richness and diversity with objective and subjective sleep measures in a population sample. *Sleep.* (2023) [Epub ahead of print]. 10.1093/sleep/zsad300 37988614 PMC10926107

[86] Bana B Cabreiro F. The microbiome and aging. *Annu Rev Genet.* (2019) 53:239–61. 10.1146/annurev-genet-112618-043650 31487470

[87] Lopez-Otin C Blasco M Partridge L Serrano M Kroemer G. Hallmarks of aging: An expanding universe. *Cell.* (2023) 186:243–78. 10.1016/j.cell.2022.11.001 36599349

[88] Leite G Pimentel M Barlow G Chang C Hosseini A Wang J Age and the aging process significantly alter the small bowel microbiome. *Cell Rep.* (2021) 36:109765. 10.1016/j.celrep.2021.109765 34592155

[89] Matenchuk B Mandhane P Kozyrskyj A. Sleep, circadian rhythm, and gut microbiota. *Sleep Med Rev.* (2020) 53:101340. 10.1016/j.smrv.2020.101340 32668369

[90] Landete J Gaya P Rodriguez E Langa S Peiroten A Medina M Probiotic bacteria for healthier aging: immunomodulation and metabolism of phytoestrogens. *Biomed Res Int.* (2017) 2017:5939818. 10.1155/2017/5939818 29109959 PMC5646295

[91] Cui X Wang L Zuo P Han Z Fang Z Li W D-galactose-caused life shortening in Drosophila melanogaster and *Musca domestica* is associated with oxidative stress. *Biogerontology.* (2004) 5:317–25. 10.1007/s10522-004-2570-3 15547319

[92] Oyebode O Abolaji A Oluwadare J Adedara A Olorunsogo O. Apigenin ameliorates D-galactose-induced lifespan shortening effects via antioxidative activity and inhibition of mitochondrial-dependent apoptosis in Drosophila melanogaster. *J Funct Foods.* (2020) 69:103957. 10.1016/j.jff.2020.103957

[93] Elkhedir A Iqbal A Zogona D Mohammed H Murtaza A Xu X. Apigenin glycosides from green pepper enhance longevity and stress resistance in *Caenorhabditis elegans*. *Nutr Res.* (2022) 102:23–34. 10.1016/j.nutres.2022.02.003 35366456

[94] Cheng Y Hou B Xie G Shao Y Yang J Xu C. Transient inhibition of mitochondrial function by chrysin and apigenin prolong longevity via mitohormesis in *C. elegans*. *Free Radic Biol Med.* (2023) 203:24–33. 10.1016/j.freeradbiomed.2023.03.264 37023934

[95] Nicholas C Batra S Vargo M Voss O Gavrilin M Wewers M Apigenin blocks lipopolysaccharide-induced lethality in vivo and proinflammatory cytokines expression by inactivating NF-kappaB through the suppression of p65 phosphorylation. *J Immunol.* (2007) 179:7121–7. 10.4049/jimmunol.179.10.7121 17982104

[96] Panda S Kar A. Apigenin (4’,5,7-trihydroxyflavone) regulates hyperglycaemia, thyroid dysfunction and lipid peroxidation in alloxan-induced diabetic mice. *J Pharm Pharmacol.* (2007) 59:1543–8. 10.1211/jpp.59.11.0012 17976266

[97] Escande C Nin V Price N Capellini V Gomes A Barbosa M Flavonoid apigenin is an inhibitor of the NAD+ ase CD38: implications for cellular NAD+ metabolism, protein acetylation, and treatment of metabolic syndrome. *Diabetes.* (2013) 62:1084–93. 10.2337/db12-1139 23172919 PMC3609577

[98] Mahajan U Chandrayan G Patil C Arya D Suchal K Agrawal Y The protective effect of apigenin on myocardial injury in diabetic rats mediating activation of the PPAR-gamma Pathway. *Int J Mol Sci.* (2017) 18:756. 10.3390/ijms18040756 28375162 PMC5412341

[99] Li H Chen D Zhang X Chen M Zhi Y Cui W Screening of an FDA-approved compound library identifies apigenin for the treatment of myocardial injury. *Int J Biol Sci.* (2023) 19:5233–44. 10.7150/ijbs.85204 37928261 PMC10620826

[100] Lu Z Liu L Zhao S Zhao J Li S Li M. Apigenin attenuates atherosclerosis and non-alcoholic fatty liver disease through inhibition of NLRP3 inflammasome in mice. *Sci Rep.* (2023) 13:7996. 10.1038/s41598-023-34654-2 37198205 PMC10192453

[101] Cavalier A Clayton Z Wahl D Hutton D McEntee C Seals D Protective effects of apigenin on the brain transcriptome with aging. *Mech Ageing Dev.* (2024) 217:111889. 10.1016/j.mad.2023.111889 38007051 PMC10843586

[102] Sudhakaran M Navarrete T Mejia-Guerra K Mukundi E Eubank T Grotewold E Transcriptome reprogramming through alternative splicing triggered by apigenin drives cell death in triple-negative breast cancer. *Cell Death Dis.* (2023) 14:824. 10.1038/s41419-023-06342-6 38092740 PMC10719380

[103] Ohrnberger J Fichera E Sutton M. The relationship between physical and mental health: a mediation analysis. *Soc Sci Med.* (2017) 195:42–9. 10.1016/j.socscimed.2017.11.008 29132081

[104] Fan X Fan Z Yang Z Huang T Tong Y Yang D Flavonoids-natural gifts to promote health and longevity. *Int J Mol Sci.* (2022) 23:2176. 10.3390/ijms23042176 35216290 PMC8879655

[105] Hano C Tungmunnithum D. Plant polyphenols, more than just simple natural antioxidants: oxidative stress, aging and age-related diseases. *Medicines.* (2020) 7:26. 10.3390/medicines7050026 32397520 PMC7281114

[106] Gilmour B Gudmundsrud R Frank J Hov A Lautrup S Aman Y Targeting NAD(+) in translational research to relieve diseases and conditions of metabolic stress and ageing. *Mech Ageing Dev.* (2020) 186:111208. 10.1016/j.mad.2020.111208 31953124

[107] Massudi H Grant R Braidy N Guest J Farnsworth B Guillemin G. Age-associated changes in oxidative stress and NAD+ metabolism in human tissue. *PLoS One.* (2012) 7:e42357. 10.1371/journal.pone.0042357 22848760 PMC3407129

[108] Zhu X Lu M Lee B Ugurbil K Chen W. In vivo NAD assay reveals the intracellular NAD contents and redox state in healthy human brain and their age dependences. *Proc Natl Acad Sci U S A.* (2015) 112:2876–81. 10.1073/pnas.1417921112 25730862 PMC4352772

[109] Camacho-Pereira J Tarrago M Chini C Nin V Escande C Warner G CD38 Dictates Age-Related NAD Decline and Mitochondrial Dysfunction through an SIRT3-Dependent Mechanism. *Cell Metab.* (2016) 23:1127–39. 10.1016/j.cmet.2016.05.006 27304511 PMC4911708

[110] Radenkovic D Reason, Verdin E. Clinical Evidence for Targeting NAD Therapeutically. *Pharmaceuticals.* (2020) 13:247. 10.3390/ph13090247 32942582 PMC7558103

[111] Fang E Lautrup S Hou Y Demarest T Croteau D Mattson M NAD(+) in Aging: Molecular Mechanisms and Translational Implications. *Trends Mol Med.* (2017) 23:899–916. 10.1016/j.molmed.2017.08.001 28899755 PMC7494058

[112] Janssens G Grevendonk L Perez R Schomakers B de Vogel-van den Bosch J Geurts J. Healthy aging and muscle function are positively associated with NAD(+) abundance in humans. *Nat Aging.* (2022) 2:254–63. 10.1038/s43587-022-00174-3 37118369

[113] Fukuwatari T Shibata K Ishihara K Fushiki T Sugimoto E. Elevation of blood NAD level after moderate exercise in young women and mice. *J Nutr Sci Vitaminol.* (2001) 47:177–9. 10.3177/jnsv.47.177 11508711

[114] Lamb D Moore J Mesquita P Smith M Vann C Osburn S Resistance training increases muscle NAD(+) and NADH concentrations as well as NAMPT protein levels and global sirtuin activity in middle-aged, overweight, untrained individuals. *Aging.* (2020) 12:9447–60. 10.18632/aging.103218 32369778 PMC7288928

[115] van de Weijer T Phielix E Bilet L Williams E Ropelle E Bierwagen A Evidence for a direct effect of the NAD+ precursor acipimox on muscle mitochondrial function in humans. *Diabetes.* (2015) 64:1193–201. 10.2337/db14-0667 25352640 PMC4375076

[116] Fukamizu Y Uchida Y Shigekawa A Sato T Kosaka H Sakurai T. Safety evaluation of beta-nicotinamide mononucleotide oral administration in healthy adult men and women. *Sci Rep.* (2022) 12:14442. 10.1038/s41598-022-18272-y 36002548 PMC9400576

[117] Igarashi M Nakagawa-Nagahama Y Miura M Kashiwabara K Yaku K Sawada M Chronic nicotinamide mononucleotide supplementation elevates blood nicotinamide adenine dinucleotide levels and alters muscle function in healthy older men. *NPJ Aging.* (2022) 8:5. 10.1038/s41514-022-00084-z 35927255 PMC9158788

[118] Yoshino M Yoshino J Kayser B Patti G Franczyk M Mills K Nicotinamide mononucleotide increases muscle insulin sensitivity in prediabetic women. *Science.* (2021) 372:1224–9. 10.1126/science.abe9985 33888596 PMC8550608

[119] Kim M Seol J Sato T Fukamizu Y Sakurai T Okura T. Effect of 12-week intake of nicotinamide mononucleotide on sleep quality, fatigue, and physical performance in older Japanese adults: a randomized, double-blind placebo-controlled study. *Nutrients.* (2022) 14:755. 10.3390/nu14040755 35215405 PMC8877443

[120] Pencina K Valderrabano R Wipper B Orkaby A Reid K Storer T Nicotinamide adenine dinucleotide augmentation in overweight or obese middle-aged and older adults: a physiologic study. *J Clin Endocrinol Metab.* (2023) 108:1968–80. 10.1210/clinem/dgad027 36740954 PMC11491622

[121] Akasaka H Nakagami H Sugimoto K Yasunobe Y Minami T Fujimoto T Effects of nicotinamide mononucleotide on older patients with diabetes and impaired physical performance: A prospective, placebo-controlled, double-blind study. *Geriatr Gerontol Int.* (2023) 23:38–43. 10.1111/ggi.14513 36443648

[122] Yi L Maier A Tao R Lin Z Vaidya A Pendse S The efficacy and safety of beta-nicotinamide mononucleotide (NMN) supplementation in healthy middle-aged adults: a randomized, multicenter, double-blind, placebo-controlled, parallel-group, dose-dependent clinical trial. *Geroscience.* (2023) 45:29–43. 10.1007/s11357-022-00705-1 36482258 PMC9735188

[123] Dollerup O Christensen B Svart M Schmidt M Sulek K Ringgaard S A randomized placebo-controlled clinical trial of nicotinamide riboside in obese men: safety, insulin-sensitivity, and lipid-mobilizing effects. *Am J Clin Nutr.* (2018) 108:343–53. 10.1093/ajcn/nqy132 29992272

[124] Trammell S Schmidt M Weidemann B Redpath P Jaksch F Dellinger R Nicotinamide riboside is uniquely and orally bioavailable in mice and humans. *Nat Commun.* (2016) 7:12948. 10.1038/ncomms12948 27721479 PMC5062546

[125] Elhassan Y Kluckova K Fletcher R Schmidt M Garten A Doig C Nicotinamide riboside augments the aged human skeletal muscle NAD(+) metabolome and induces transcriptomic and anti-inflammatory signatures. *Cell Rep.* (2019) 28:1717–28.e6. 10.1016/j.celrep.2019.07.043 31412242 PMC6702140

[126] Ogura Y Kitada M Xu J Monno I Koya D. CD38 inhibition by apigenin ameliorates mitochondrial oxidative stress through restoration of the intracellular NAD(+)/NADH ratio and Sirt3 activity in renal tubular cells in diabetic rats. *Aging.* (2020) 12:11325–36. 10.18632/aging.103410 32507768 PMC7343471

